# Reliability and Validity of Measuring the Strength of the Chin-Tuck Maneuver in Community-Dwelling Older Adults as a Means of Evaluating Swallowing-Related Muscle Strength

**DOI:** 10.3390/geriatrics9060148

**Published:** 2024-11-13

**Authors:** Naoto Kamide, Takeshi Murakami, Masataka Ando, Takuya Sawada, Wakana Hata, Miki Sakamoto

**Affiliations:** 1 School of Allied Health Sciences, Kitasato University, Kitazato 1-15-1, Minami-ku, Sagamihara 252-0373, Japan; takeshim@kitasato-u.ac.jp (T.M.); m.ando@kitasato-u.ac.jp (M.A.); sawada.takuya2@kitasato-u.ac.jp (T.S.); hata-w@kitasato-u.ac.jp (W.H.); mikis@kitasato-u.ac.jp (M.S.); 2 Graduate School of Medical Sciences, Kitasato University, Kitazato 1-15-1, Minami-ku, Sagamihara 252-0373, Japan

**Keywords:** chin-tuck maneuver, older people, reliability, sarcopenia, swallowing-related muscle function, suprahyoid muscle, tongue pressure, validity

## Abstract

**Background**: The chin-tuck maneuver has been suggested to increase suprahyoid muscle activation, but a method to measure the strength of the chin-tuck maneuver has not been established. We developed a method to measure the strength of the chin-tuck maneuver (chin-tuck strength) and examined the reliability and validity of chin-tuck-strength measurement in community-dwelling older adults. **Participants and Methods**: The participants were 233 older adults aged ≥65 years without dysphagia or physical disability. Chin-tuck strength was measured twice consecutively using the developed device, and reproducibility was analyzed using intraclass correlation coefficients (ICCs). In addition, maximum tongue pressure, oral diadochokinesis, grip strength, knee extension strength, and the timed up and go test (TUGT) were measured as indices of swallowing-related muscle function and appendicular muscle function. The associations of chin-tuck strength with swallowing-related muscle function and appendicular muscle function were analyzed statistically. **Results**: The ICCs for chin-tuck strength were 0.82 (95% confidence interval [CI]: 0.73–0.88) in males and 0.87 (95% CI: 0.70–0.93) in females. Chin-tuck strength was significantly associated with maximum tongue pressure, grip strength, knee extension strength, and TUGT. **Conclusions**: This study suggests that chin-tuck strength is a reliable and valid assessment of swallowing-related muscle strength.

## 1. Introduction

Dysphagia in older people is a well-known risk factor for aspiration pneumonia and poor quality of life [1]. Dysphagia is not only caused by neurological diseases such as stroke and neurodegenerative diseases but also by sarcopenia in older people [2,3]. Dysphagia caused by sarcopenia has recently been defined as sarcopenic dysphagia, which is swallowing dysfunction due to sarcopenia in both appendicular skeletal muscles and swallowing-related muscles [3]. Sarcopenia is now widely recognized as a common health problem, with a systematic review suggesting that approximately 10% of community-dwelling older people have sarcopenia [4]. Similarly, a recent meta-analysis of dysphagia in older people suggested that approximately 20% of community-dwelling older people may have dysphagia [5]. Therefore, dysphagia in older people associated with sarcopenia should be recognized by health professionals as a common health problem that requires care and prevention.

Assessment of swallowing-related muscle function is essential for the management and prevention of sarcopenic dysphagia. The published diagnostic criteria for sarcopenic dysphagia [3] and oral hypofunction [6] have proposed tongue pressure measurement to assess swallowing-related muscle strength. Tongue pressure measurement has been reported to be associated with sarcopenia [7], appendicular skeletal muscle mass and strength [8,9], and geniohyoid and pharyngeal muscle mass [10]. It has also been suggested that middle-aged and older people with aspiration pneumonia may have reduced tongue pressure [11]. Therefore, it may be reasonable to assess tongue pressure as an index of swallowing-related muscle strength. However, tongue pressure measures the strength of the tongue, not the strength of the suprahyoid muscle group directly, which is involved in opening the pharyngoesophageal segment [12] and may contribute to improved swallowing function [13].

The chin-tuck-against-resistance exercise has recently been reported as an effective therapeutic exercise for dysphagia rehabilitation [14]. The chin-tuck maneuver involves tucking the chin toward the manubrium sterni, and adding isometric or isokinetic resistance to the chin-tuck maneuver has been reported to increase suprahyoid muscle activation [15]. In addition, previous studies of the chin-tuck-against-resistance exercise have reported improvements in tongue pressure [16] and swallowing function [14]. Therefore, measuring the strength of the chin-tuck maneuver (chin-tuck strength) may be an effective and reasonable assessment of swallowing-related muscle strength. However, the reliability of measurement and the validity of chin-tuck strength as an assessment of swallowing-related muscle strength have not been clarified in previous studies.

In this study, we hypothesized that chin-tuck strength can be measured reliably, and that with respect to validity, chin-tuck strength is associated with swallowing-related muscle strength such as tongue pressure, whole-body muscle function, and skeletal muscle mass. Therefore, the aim of this study was to examine the reliability of chin-tuck-strength measurement and its associations with tongue pressure, whole-body muscle function, and skeletal muscle mass in community-dwelling older adults.

## 2. Materials and Methods

### 2.1. Participants

The participants were 233 community-dwelling older adults aged ≥65 years. They were volunteers recruited through local newspapers in Sagamihara City, Japan. Recruitment and data collection took place between August 2023 and February 2024. Inclusion criteria were as follows: age ≥65 years, independence in activities of daily living (ADL), and without restriction of oral intake. Institutionalized older adults, those younger than 65 years, those with disability in ADL, those with a history of stroke, those with Parkinson’s disease, those with cervical myelopathy and cervical disc herniation, and those with neck pain were excluded from this study. Independence in ADL was confirmed by the certification of care need levels for long-term care insurance in Japan [17], and those with any care need level were excluded. Restriction of oral intake was confirmed using the functional oral intake scale (FOIS) [18], and those with an FOIS level of less than 7 were excluded. The presence of a history of stroke or Parkinson’s disease was confirmed by a self-administered questionnaire. The presence of cervical myelopathy, cervical disc herniation, and neck pain was confirmed in a face-to-face interview by trained researchers.

This study was approved by the institutional review board of the School of Allied Health Sciences at Kitasato University (Approval No. 2023-008). Written, informed consent was obtained from all participants.

### 2.2. Device for Measuring Chin-Tuck Strength

**Figure 1:**
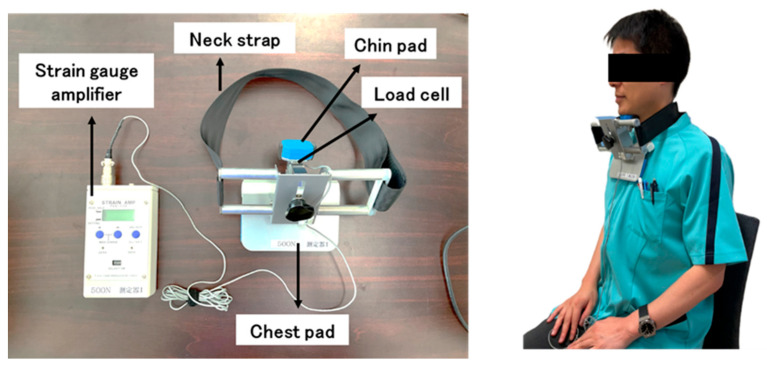
The **left** image shows the device developed in this study, consisting of a strain gauge amplifier, neck strap, chin and chest pad, and load cell. The **right** image shows a participant wearing the device. Participants were asked to rest their back against the backrest of the chair and tuck their chin toward the manubrium sterni with maximum isometric contraction.

The device shown on the left side of Figure 1 was developed in the present study to measure chin-tuck strength. It consisted of a strain gauge amplifier (T.K.K. 1268, SANKA Co., Ltd., Niigata, Japan) and an instrument for holding the neck with a chin pad, chest pad, and neck strap, and the load cell (TU-MXR2(T)500N-G3, TEAC Co., Ltd., Tokyo, Japan) was embedded under the chin pad of the instrument. Participants attached the instrument to the front of their neck with the chin pad and chest pad positioned at the chin and sternum, respectively, while sitting on a chair with a backrest. It was secured with the neck strap at the neutral position of the neck (flexion and extension at zero degrees) (Figure 1, right).

Participants were asked to rest their back against the backrest of the chair and tuck their chin toward the manubrium sterni with a maximal isometric contraction for 5 s. The maximum force generated during the chin-tuck maneuver was recorded as chin-tuck strength using the measuring device. Chin-tuck strength was measured two consecutive times with a 30 s rest period. Then, the two measurements were used for reproducibility analysis, and the mean of the two measurements was used for validity analysis.

### 2.3. Swallowing-Related Muscle Function

Maximum tongue pressure (MTP) was measured as an index of swallowing-related muscle strength, and oral diadochokinesis (ODK) was measured as an index of lip–tongue motor function [6]. MTP data were collected using a tongue pressure measuring device with a disposable probe inserted into the oral cavity on the anterior side of the tongue (TPM-01, JMS Co., Ltd., Hiroshima, Japan) [19], and measurements were taken three consecutive times with a 30 s rest period. The mean of the three measurements was used for further statistical analysis. In addition, tongue pressure less than 30 kPa was defined as low tongue pressure according to the diagnostic criteria for oral hypofunction [7]. ODK was assessed by three different sounds (“pa”, “ta”, and “ka”) using a device (T.K.K.3351, SANKA Co., Ltd., Niigata, Japan). Participants were asked to produce each sound for 5 s, and the number of sounds produced per second for each sound was recorded.

### 2.4. Muscle Strength of the Upper and Lower Limbs, Skeletal Muscle Mass, and Physical Performance

Muscle strength of the upper and lower limbs was measured by grip strength and knee extension strength, respectively. Grip strength was measured in a standing position with the dominant hand using a Smedley-type dynamometer (T.K.K.5401, SANKA Co., Ltd.). Knee extension strength was measured using a handheld dynamometer (μ-Tas F-1; Anima Inc., Tokyo, Japan) on the right side in a sitting position with the knee and hip joint flexed at 90 degrees.

Appendicular skeletal muscle mass was measured using a bioimpedance analysis method (InBody 430; InBody Japan Inc., Tokyo, Japan). Appendicular skeletal muscle mass was divided by the square of the body height, and the appendicular skeletal muscle mass index (SMI) was calculated and used for further statistical analysis.

For the timed up and go test (TUGT), participants were asked to stand up from a chair without hand support, walk 3 m as quickly as possible, turn around, walk back, and then sit down again without hand support [20], and the time required to complete this task was measured.

### 2.5. Covariates

The body mass index (BMI), number of prescribed medications, and number of comorbidities were included as covariates. Height and weight were measured using a height rod and weight scale, respectively, and BMI was calculated. The number of prescribed medications and number of comorbidities were confirmed using a self-administered questionnaire. For the number of prescribed medications, participants were asked about the number of types of oral medications prescribed by the physician. For the number of comorbidities, they were asked if a doctor had diagnosed any of the following diseases: hypertension, diabetes mellitus, cancer, chronic lung disease, heart attack, congestive heart failure, angina, asthma, arthritis, stroke, Parkinson’s disease, and kidney disease. Those who responded that they had stroke or Parkinson’s disease were excluded from this study. The number of other diseases they had was counted and used in the analysis.

### 2.6. Statistical Analysis

To verify reliability, the reproducibility of the two measurements of chin-tuck strength was assessed by the intraclass correlation coefficient (ICC) with a two-way mixed model based on single measurement. An ICC of 0.75 or more was then defined as good reliability [21]. For validity, the relationships between chin-tuck strength and MTP, ODK, grip strength, knee extension strength, and SMI were examined using Pearson’s product-moment correlation coefficient and multiple regression analysis, with chin-tuck strength as the dependent variable. In addition, to assess potential overfitting of the multiple regression model, the least absolute shrinkage and selection operator (LASSO) model was applied with 10-fold cross-validation, and the regression coefficients were estimated. Statistical analysis was performed using R Statistical Analysis Software Version 4.0.3 (R Foundation for Statistical Computing, Vienna, Austria), with significance set at 5%.

## 3. Results

### 3.1. Characteristics of the Participants

The basic characteristics of the participants and the descriptive statistics of the variables measured in this study are presented in Table 1.

### 3.2. The Reliability of Chin-Tuck-Strength Measurements

For the reproducibility of chin-tuck-strength measurements, the ICC was 0.890 (95% confidence interval [CI]: 0.826–0.926) for the total sample, 0.817 (95% CI: 0.726–0.879) for males, and 0.865 (95% CI: 0.704–0.927) for females.

### 3.3. The Validity of Chin-Tuck-Strength Measurements

**Figure 2:**
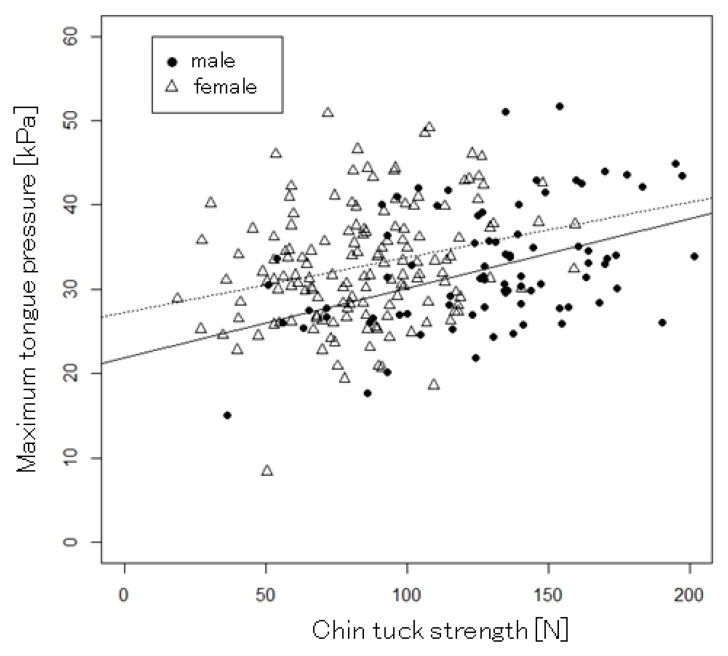
In the scatter plot, the black circles represent male participants, and the white triangles represent female participants. The solid line is the regression line between chin-tuck strength and tongue pressure for male participants, and the dashed line is that for female participants.

**Figure 3:**
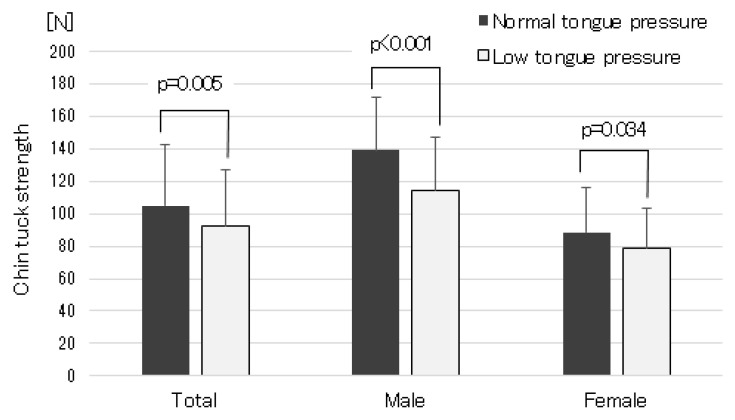
The black bar represents chin-tuck strength in participants with normal tongue pressure (≥30 kPa), and the gray bar represents chin-tuck strength in participants with low tongue pressure (<30 kPa).

The results of the correlation analysis between chin-tuck strength and swallowing-related muscle functions and upper and lower limb muscle functions are presented in Table 2. Furthermore, for the relationship between chin-tuck strength and MTP, which is an index of swallowing-related muscle strength, the scatter plots for male and female participants are shown in Figure 2. Chin-tuck strength was significantly related to MTP, grip strength, knee extension strength, SMI, and TUGT. Furthermore, these relationships remained significant when the analysis was stratified by sex. For MTP, even when MTP data were treated as categorical variables (low tongue pressure or normal tongue pressure), subjects with low tongue pressure had significantly weaker chin-tuck strength than those with normal tongue pressure (Figure 3). However, significant correlations between chin-tuck strength and ODK were not found, except for the correlation between chin-tuck strength and ODK of “ka” in females. For the covariates, chin-tuck strength was significantly related to BMI in the total sample (r = 0.257, *p* < 0.001), in males (r = 0.263, *p* < 0.001), and in females (r = 0.215, *p* < 0.001). In addition, in females only, significant relationships were found between chin-tuck strength and age (r = −0.21, *p* = 0.01) and number of prescribed medications (r = 0.16, *p* = 0.046). However, these correlations were weak overall. No significant relationships were found between chin-tuck strength and number of comorbidities in the total sample, in males, and in females.

Finally, multiple regression analysis adjusted for age and sex was performed to determine the associations of chin-tuck strength with MTP, SMI, and upper and lower extremity muscle function. Chin-tuck strength was significantly associated with MTP, grip strength, knee extension strength, and TUGT (Table 3). In addition, as a sensitivity analysis, only MTP was treated as a categorical variable (low and normal tongue pressure) and entered into the regression model. All significant associations remained unchanged (the coefficient of MTP with low tongue pressure was −10.5, with *p* = 0.03). Furthermore, as a result of the LASSO model, the regression coefficients for all dependent variables, including tongue pressure, remained nearly unchanged (Table 3).

## 4. Discussion

Recently, it has been reported that the chin-tuck-against-resistance exercise is effective for dysphagia rehabilitation [14]. Therefore, measuring the muscle strength of the chin-tuck maneuver may be useful as an indicator of muscle strength of swallowing-related muscles, but the method for measuring chin-tuck strength has not been established. In this study, a method for measuring chin-tuck strength was developed, and the reliability and validity of chin-tuck-strength measurement were examined in community-dwelling older adults.

In terms of reliability, the measurement reproducibility of chin-tuck strength obtained in the present study was examined using the ICC, and it was found to range from 0.82 to 0.89. Therefore, this result showed that the chin-tuck strength measured in the present study had good reliability [21]. In previous studies, the ICC of tongue pressure was reported to be from 0.96 to 0.99 [22], and the ICC of physical function tests, such as grip strength, knee extension strength, and TUG, was from 0.88 to 0.96 [23]. Therefore, the method of measuring chin-tuck strength in the present study appeared to have comparable results to the widely used swallowing-related muscle strength and physical function tests.

Regarding the validity of chin-tuck-strength measurement, chin-tuck strength in the present study was significantly associated with MTP. Since MTP has been reported to be associated with the muscle thickness of the geniohyoid muscle [10], it is used clinically as an index of swallowing-related muscle strength. Furthermore, MTP has also been reported to be associated with whole-body sarcopenia [7], appendicular skeletal muscle mass [8], and grip strength [9], suggesting that MTP reflects not only swallowing-related muscle strength but also whole-body muscle mass and strength. The finding that chin-tuck strength was associated with MTP suggested that chin-tuck strength has validity as an index of swallowing-related muscle strength. In particular, the chin-tuck maneuver was previously reported to increase muscle activity of the suprahyoid muscles [15]. Therefore, it is assumed that chin-tuck strength in the present study reflects the strength of the suprahyoid muscles involved in opening the pharyngoesophageal segment during swallowing [12]. In addition, multiple regression analysis also showed that chin-tuck strength was associated with MTP independently of physical functions such as grip strength, knee extension strength, and TUGT. This result indicated the robustness of the association between chin-tuck strength and MTP.

In addition to the association with MTP, chin-tuck strength was also associated with grip strength, knee extension strength, TUGT, and SMI. These findings suggest that the chin-tuck strength obtained in the present study has validity as an assessment of muscle strength in physical functions. Therefore, chin-tuck strength was shown to be related to whole-body muscle functions and skeletal muscle mass, which was suggested to be a characteristic similar to tongue pressure. However, chin-tuck strength was not related to ODK. ODK is generally an assessment of the frequency and consistency of repetitive movements of the oral articulators [24] rather than an indicator of muscle strength. Because chin-tuck strength was considered an index of swallowing-related muscle strength, it may not be related to ODK, which is an index of oral articulator function.

As assessments of suprahyoid muscle function, measurement of muscle thickness by ultrasound [10] and muscle activity by surface electromyography [25,26] have been reported. However, measurement of muscle thickness by ultrasound generally requires a skilled examiner, and it cannot directly assess the strength of the suprahyoid muscle. Surface electromyography measurements are affected by noise interference, and it may be difficult to obtain quality data in subjects with loose skin and subcutaneous tissue, which is observed in older adults [26]. Furthermore, similar to ultrasound, surface electromyography cannot directly assess the strength of the suprahyoid muscle. The assessment of chin-tuck strength in the present study may be more convenient than suprahyoid muscle assessment by ultrasound and electromyography. In addition, we believe that the results of the present study, which demonstrated the reliability and validity of chin-tuck-strength measurement as an assessment of suprahyoid muscle strength, are novel.

The present study had several limitations that should be considered when interpreting the results. First, the reliability of chin-tuck-strength measurement was only examined for the reproducibility of two measurements. Therefore, the examination of reliability may not be sufficient. Further investigation of reliability, such as inter-rater reliability, is required. Second, the chin-tuck strength measured in this study is assumed to assess suprahyoid muscle strength. However, muscle activity and contraction were not directly assessed during chin-tuck-strength measurement in this study, and assessment of muscle activity and contraction during chin-tuck-strength measurement using surface electromyography is needed. Third, participants in this study were recruited from community-dwelling older adults who did not have restriction of oral intake. However, validated assessments for dysphagia, such as videofluoroscopic and videoendoscopic examinations of swallowing, were not possible. Therefore, the association between chin-tuck strength and dysphagia could not be clearly demonstrated, and further studies are needed.

## 5. Conclusions

This study developed a method for measuring the strength of the chin-tuck maneuver in older adults. The chin-tuck strength obtained in this study had good reliability. In addition, chin-tuck strength was associated with tongue pressure, whole-body muscle functions, and skeletal muscle mass. Therefore, chin-tuck strength had validity as an index of swallowing-related muscle strength. Further studies are needed, but the chin-tuck-strength measurement method developed in this study may be a simple and useful assessment of swallowing-related muscle strength in older adults.

## 6. Patents

Dr. Kamide and Dr. Murakami are the inventors of the chin-tuck-strength measuring device (Patent No. 7495133), which is registered with the Japan Patent Office.

## Figures and Tables

**Table 1:** Basic characteristics of the participants and descriptive statistics.

	Total	Male	Female
	*n* = 233	*n* = 81	*n* = 152
	Mean (SD)	Mean (SD)	Mean (SD)
Age (y)	74.2 (5.2)	75.4 (5.6)	73.6 (4.9)
Body mass index (kg/m^2^)	22.3 (3.0)	22.9 (2.4)	22.0 (3.3)
No. of prescribed medicines	2.0 (2.1)	2.4 (2.5)	1.8 (1.8)
No. of comorbidities	1.1 (0.9)	1.3 (1.0)	1.0 (0.9)
Grip strength (kg)	27.2 (6.4)	33.8 (5.1)	23.7 (3.6)
Knee extension strength (kg)	25.8 (8.2)	30.0 (8.2)	23.6 (7.3)
Skeletal muscle mass index (kg/m^2^)	6.5 (0.9)	7.4 (0.6)	6.0 (0.6)
Timed up and go test (s)	5.7 (1.0)	5.6 (1.0)	5.8 (0.9)
ODK ^1^ [pa] (times/s)	6.5 (0.7)	6.4 (0.6)	6.6 (0.7)
ODK ^1^ [ta] (times/s)	6.4 (0.7)	6.2 (0.7)	6.5 (0.7)
ODK ^1^ [ka] (times/s)	5.9 (0.7)	5.6 (0.7)	6.1 (0.6)
Maximum tongue pressure (kPa)	32.7 (7.0)	32.6 (7.1)	32.8 (6.9)
Chin-tuck strength (N)	100.4 (37.2)	129.5 (36.0)	84.9 (27.4)

^1^ ODK, oral diadochokinesis. SD, standard deviation.

**Table 2:** Correlations between chin-tuck strength and swallowing-related muscle functions and upper and lower limb muscle functions.

	Sample	Coefficient	*p* Value
Maximum tongue pressure (kPa)	Total	0.256	<0.001
	Male	0.418	<0.001
	Female	0.256	0.002
ODK ^1^ [pa] (times/s)	Total	−0.017	0.795
	Male	0.069	0.544
	Female	0.069	0.400
ODK ^1^ [ta] (times/s)	Total	−0.038	0.564
	Male	0.136	0.228
	Female	0.133	0.103
ODK ^1^ [ka] (times/s)	Total	−0.134	0.042
	Male	0.045	0.689
	Female	0.110	0.181
Skeletal muscle mass index (kg/m^2^)	Total	0.603	<0.001
	Male	0.326	0.003
	Female	0.335	<0.001
Grip strength (kg)	Total	0.637	<0.001
	Male	0.388	<0.001
	Female	0.377	<0.001
Knee extension strength (kg)	Total	0.562	<0.001
	Male	0.517	<0.001
	Female	0.412	<0.001
Timed up and go test (s)	Total	−0.333	<0.001
	Male	−0.365	<0.001
	Female	−0.360	<0.001

^1^ ODK, oral diadochokinesis. Coefficients calculated using Pearson’s product-moment correlation.

**Table 3:** Associations of chin-tuck strength with tongue pressure and upper and lower limb muscle function.

	Coefficient	SE ^1^	*p* Value	Coefficient by LASSO ^2^
Maximum tongue pressure (kPa)	0.86	0.25	0.001	0.85
Grip strength (kg)	1.39	0.47	0.003	1.39
Knee extension strength (kg)	1.21	0.25	<0.001	1.21
Skeletal muscle mass index	2.51	3.40	0.462	2.52
Timed up and go test (s)	−6.12	1.90	0.001	−6.10
Age (y)	0.20	0.36	0.576	0.19
Sex (female)	−18.55	6.12	0.003	−18.52

Dependent variable: chin-tuck strength. Coefficients are unstandardized coefficients by multiple regression analysis. ^1^ SE, standard error; ^2^ coefficient by LASSO, regression coefficients estimated by the least absolute shrinkage and selection operator.

## Data Availability

The datasets generated and analyzed during the current study are available from the corresponding author upon reasonable request.
